# Supplementary material for: Using the C-Read as a Portable Device to Evaluate Reading Ability in Young Chinese Adults: An Observational Study
Source: J Pers Med. 2023 Mar 1;13(3):463. doi: 10.3390/jpm13030463 (PMC10056310; doi:10.3390/jpm13030463)
Supplement: Supplementary file 1 [file jpm-13-00463-s001.zip › C-READ reading ability test questionnaire.pdf]

C-READ reading ability test

You have volunteered for this research project [single-choice topic] *

○ Yes

○ No (please jump to the end of the questionnaire and submit the answer sheet)

1. How do you feel about your health overall ()

○ a. excellent ○ b. good ○ c. good D. fair ○ e. poor

2. What is the current visual acuity (corrected visual acuity) of your eyes? () [single topic] *

○ a. excellent ○ b. good ○ c. fair

○ d. poor ○ e. very poor ○ f. completely invisible

3. Do you often worry about your vision condition? () [single topic] *

A. never worry B. a little worried C. sometimes worried D. most of the time worried E. always worried

4. Is there any pain or discomfort (such as burning sensation, itching or pain) in or around your eyes? [single topic] *

○ a. no ○ b. minor ○ c. moderate ○ d. heavier ○ e. very serious

5. How difficult is it for you to read ordinary fonts in the newspaper? () [single topic] *

○ a. no difficulties ○ b. a little bit difficult ○ c. difficulties (moderate)

D. it is indeed difficult E. no longer reading due to vision ○ f. not reading newspapers due to other reasons or lack of interest

6. How difficult is it for you to do something that needs to be seen more clearly (for example, cooking, sewing, nailing around a room or using tools)? () [single topic] *

○ a. no difficulties ○ b. a little bit difficult ○ c. difficulties (moderate)

D. it is indeed difficult ○ e. has not done so due to vision reasons ○ f. not doing it for other reasons or not interested

7. How much difficulty do you have in finding things on crowded shelves or shelves due to vision? () [single topic] *

○ a. no difficulties ○ b. a little bit difficult ○ c. difficulties (moderate)

D. it is indeed difficult	○ e. has not done so due to vision reasons	○ f. not doing it for other reasons or not interested

8. How hard do you have to see street signs or store names on the street? () [single topic] *

○ a. no difficulties	○ b. a little bit difficult	○ c. difficulties (moderate)
D. it is indeed difficult	○ e. has not done so due to vision reasons	○ f. not doing it for other reasons or not interested

9. Due to poor eyesight, how difficult is it for you to walk down the stairs or steps in dim light or at night? [single topic] *

○ a. no difficulties	○ b. a little bit difficult	○ c. difficulties (moderate)
D. it is indeed difficult	○ e. has not done so due to vision reasons	○ f. not doing it for other reasons or not interested

10. Due to your eyesight, how difficult are you to see clearly the things beside the objects on the road when you walk along the street? () [single topic] *

○ a. no difficulties	○ b. a little bit difficult	○ c. difficulties (moderate)
D. it is indeed difficult	○ e. has not done so due to vision reasons	○ f. not doing it for other reasons or not interested

11. How difficult is it for you to see clearly the other party's reaction to what you have said when talking with others due to vision [single-choice topic] *

○ a. no difficulties	○ b. a little bit difficult	○ c. difficulties (moderate)
D. it is indeed difficult	○ e. has not done so due to vision reasons	○ f. not doing it for other reasons or not interested

12. How much difficulty do you have in choosing matching clothes due to vision? [single topic] *

○ a. no difficulties	○ b. a little bit difficult	○ c. difficulties (moderate)
D. it is indeed difficult	○ e. has not done so due to vision reasons	○ f. not doing it for other reasons or not interested

13. How much difficulty do you have in going to someone's house, party or restaurant due to vision? () [single topic] *

○ a. no difficulties	○ b. a little bit difficult	○ c. difficulties (moderate)
D. it is indeed difficult	○ e. has not done so due to vision reasons	○ f. not doing it for other reasons or not interested

14. How hard do you have to go to the movies, performances or sports competitions due to your eyesight? [single topic] *

○ a. no difficulties ○ b. a little bit difficult ○ c. difficulties (moderate)

D. it is indeed difficult ○ e. has not done so due to vision reasons ○ f. not doing it for other reasons or not interested

15. Have you ridden a bicycle or driven a motor vehicle (motorcycle or car) recently? [single topic] *

A. yes (please jump to question 19) B none

15A If not: Have you never ridden or have you stopped? () [single topic] *

○ a. never ride (drive) a car ○B. stop riding (driving)

15B. Do you not ride (drive) the car mainly due to your vision or other reasons, or both? () [single topic] *

○ a. mainly due to vision ○ b. other reasons C. Vision and other causes

* After completing this question, please jump to question 22.

15C If you have been riding (driving) a car recently, how much difficulty do you have in riding (driving) a car in a familiar place during the daytime? () [single topic] *

○ a. no difficulties ○ b. a little bit difficult ○ c. difficulties (moderate) D. it is indeed difficult

16. How hard do you have to ride at night? () [single topic] *

○ a. no difficulties ○ b. a little bit difficult ○ c. difficulties (moderate)

D. it is indeed difficult ○ e. has not done so due to vision reasons ○ f. not doing it for other reasons or not interested

16A. How difficult are you to ride (drive) in difficult conditions, such as bad weather, rush hours, highways, or heavy traffic? () [single topic] *

○ a. no difficulties ○ b. a little bit difficult ○ c. difficulties (moderate)

D. it is indeed difficult ○ e. has not done so due to vision reasons ○ f. not doing it for other reasons or not interested

17. Do you often fail to achieve your expected work goals due to vision issues? () [single topic] *

A, always like this B, most of the time C, sometimes it is like this D, occasionally E, no

18. Do you have limited time to work or do other things due to vision () [single-choice] *

A, always like this B, most of the time C, sometimes it is like this D, occasionally E, no

19. Do you often feel pain or discomfort in or around your eyes that affects or hinders you from doing something? () [single topic] *

A, always like this	B, most of the time	C, sometimes it is like this	D, occasionally	E, no

20. Due to my vision problem, I spend most of my time waiting for home ()

A, indeed	B, probably so	C, not sure	D, probably not	E, really not the case

21. I feel frustrated a lot of time due to my vision problem ().

A, indeed	B, probably so	C, not sure	D, probably not	E, really not the case

22. It's hard for me to control what I do due to my vision problem ().

A, indeed	B, probably so	C, not sure	D, probably not	E, really not the case

23. Due to my vision problem, I have to rely more on others to tell me a lot of things ().

A, indeed	B, probably so	C, not sure	D, probably not	E, really not the case

24. I need more help from others due to my vision problems ().

A, indeed	B, probably so	C, not sure	D, probably not	E, really not the case

25. Due to my vision problem, I am worried about doing something that will embarrass me or others ().

A, indeed	B, probably so	C, not sure	D, probably not	E, really not the case

Your Chinese name [fill in the blanks] *

____

Your age (enter an integer) [fill in the blanks] *

____

Your gender [single choice] *

○ Male

○ female

Do you have any eye conditions that affect reading (eg, glaucoma, cataracts, high myopia above 800 degrees) [single issue] *

○ Yes (please specify: _)

None

What is the degree of myopia in your left eye? [Fill in the blanks] *

____

What is the degree of myopia in your right eye? [Fill in the blanks] *

____

How long have you had an average of screen usage per day since last January? [single topic] *

A. < 2 h (electronic screen use rarely, only at a fixed time to see mobile phone computer)

B. 2-4h (mild mobile phone users who rarely use a computer)

C. 4-6 h (easy to work with a computer or normal mobile phone users)

D. 6-8h (normal working or moderate mobile phone users, such as online class)

E. 8-10 h (hard work or heavy mobile phone users, such as online class and play mobile phone for a while)

F. 10-12h (very hard work, such as playing mobile phone after a whole day's online class)

G. 12-14h (very hard work, e.g. playing mobile phone for a long time after a whole day of online lessons)

H. 14-16h (watching electronic screen except sleeping time)

I. > 16 h (nearly don't sleep)

How long have you spent on average every day using your mobile phone in the past month?

A. 0 (no mobile phone used)

B. < 2 h (only at a fixed time to see mobile phone, use very little)

C. 2-4 h (mild mobile phone users)

D. 4-6h (moderate mobile phone users)

E. 6-8 h (severe mobile phone users)

F. 8-10 h (need to use mobile phone work or very heavy mobile phone users)

G. 10-12h (need to use a mobile phone to do very hard work, such as a whole day net class and play mobile phone for a while)

H. > 12h (using mobile phone almost except sleeping time)

How long have you spent on average daily computer use in the past month?

A. 0 (no computer used)

B. 0-2h (few computers used)

C. 2-4 h (mild computer users, such as need to use the computer to complete a single task)

D. 4-6 h (normal computer users, such as need to use the computer to work)

E. 6-8h (moderate computer users, such as during the daytime need to use computer work)

F. 8-10h (severe computer users, need to use the computer to work during the daytime and at night)

G. 10-12h (need to use a computer to do very hard work, such as a whole day net class and play some games)

H. > 12h (using computer almost except sleeping time)

How long have you used your tablet or pad [single-choice] * on average per day in the last month

A. 0 (no tablet or pad used)

B. 0-2 h (mild use plate or pad)

C. 2-4 h (moderate tablet or pad using the user, such as need to use tablet or pad to complete a single task)

D. 4-6 h (moderate tablet or pad using the user, such as need to use the tablet or pad work)

E. 6-8 h (severe tablet or pad using the user, such as using a tablet or pad after play)

F. 8-10 h (severe tablet or pad users, need to use a tablet or pad during the day and night)

G.10–12h (use tablet or pad all day long, e.g. have a whole day of online lessons and play a game for a while)

H. > 12h (use tablet or pad almost except for bedtime)

Your educational background is [single-choice topic] *

A. High school and below (including reading)

B. Undergraduate course (including reading)

C. Master degree (including reading)

D. Doctor degree (including reading)

E. Postdoc (including reading)

C-READ 阅读能力测试

您自愿参与本次研究项目 [单选题] *

○是

○否 (请跳至第问卷末尾，提交答卷)

1、 总体来讲，您自我感觉自己的健康状况( ) [单选题] *

○A. 极好　○B.很好　○C.好　○D.尚可　○E.差

2、 目前您的双眼视力(矫正视力)如何？( ) [单选题] *

○A. 极好　○B.很好　○C.尚可

○D.差　○E.极差　○F.完全看不到

3、 您经常担心自己的视力状况吗？( ) [单选题] *

○A. 从不担心　○B.有一点担心　○C.有时候担心　○D.大多数时间担心　○E.一直都担心

4、 您的眼睛及眼睛周围有无无疼痛或不舒适感(如烧灼感、瘙痒或疼痛等)吗？ [单选题] *

○A. 没有　○B.轻微的　○C.中等程度的　○D.较重的　○E.很严重的

5、 您在阅读报纸上普通的字体有多大困难？( ) [单选题] *

○A. 没有困难　○B.有一点困难　○C.有困难(中等程度)

○D.的确很困难　○E.由于视力原因已不再阅读　○F.由于其他原因或没有兴趣而不阅读报纸

6、 当做一些需要看的更更清晰的事情(例例如做饭、针线活、在房间周围钉东西或者需要使用用工具)，您有多大困难？( ) [单选题] *

○A. 没有困难　○B.有一点困难　○C.有困难(中等程度)

○D.的确很困难　○E.由于视力原因已不做此事　○F.由于其他原因或没有兴趣而不做此事

7、 由于视力原因，您在拥挤的货架或书架上寻找东西时有多大困难？( ) [单选题] *

○A. 没有困难　○B.有一点困难　○C.有困难(中等程度)

○D.的确很困难 ○E.由于视力原因已不做此事 ○F.由于其他原因或没有兴趣而不做此事

8、 在街道上，您对看清街上道路标志或商店名称有多大困难？( ) [单选题] *

○A. 没有困难 ○B.有一点困难 ○C.有困难(中等程度)

○D.的确很困难 ○E.由于视力原因已不做此事 ○F.由于其他原因或没有兴趣而不做此事

9、 由于视力原因，在昏暗的灯光下或晚上，您在下楼梯、台阶时有多大困难？ [单选题] *

○A. 没有困难 ○B.有一点困难 ○C.有困难(中等程度)

○D.的确很困难 ○E.由于视力原因已不做此事 ○F.由于其他原因或没有兴趣而不做此事

10、由于视力原因，当沿街行走时，您对看清马路路上物体旁边的东西有多大困难？( ) [单选题] *

○A. 没有困难 ○B.有一点困难 ○C.有困难(中等程度)

○D.的确很困难 ○E. 由于视力原因已不做此事 ○F.由于其他原因或没有兴趣而不做此事

11、 由于视力原因，当与他人交谈时，您在看清对方对您所说事情的反应时有多大困难 [单选题] *

○A.没有困难 ○B.有一点困难 ○C.有困难(中等程度)

○D.的确很困难 ○E.由于视力原因已不做此事 ○F.由于其他原因或没有兴趣而不做此事

12、由于视力原因，您在挑选搭配衣服时有多大困难？ [单选题] *

○A.没有困难 ○B.有一点困难 ○C.有困难(中等程度)

○D.的确很困难 ○E.由于视力原因已不做此事 ○F.由于其他原因或没有兴趣而不做此事

13、由于视力原因，您在去别人家作客、参加聚会或者在餐厅就餐时有多大 困难？( ) [单选题] *

○A.没有困难 ○B.有一点困难 ○C.有困难(中等程度)

○D.的确很困难 ○E.由于视力原因已不做此事 ○F.由于其他原因或没有兴趣而不做此事

14、由于视力原因，您在外出看电影、演出或体育比赛上有多大困难？ [单选题] *

○A.没有困难 ○B.有一点困难 ○C.有困难(中等程度)

○D.的确很困难 ○E. 由于视力原因已不做此事 ○F.由于其他原因或没有兴趣而不做此事

15、您最近有没有骑自行车或驾驶机动车(摩托车、汽车)? [单选题] *

○A.有 (请跳至第 19 题) ○B 没有

15A、如果没有：您从不骑(驾)车还是您已经停止骑(驾)车了了? ( ) [单选题] *

○A.从不骑(驾)车 ○B.停止骑(驾)车

15B、您不骑(驾)车，主要是由于您的视力原因还是其他原因、抑或 视力及其它原因均有? ( ) [单选题] *

○A. 主要是视力原因 ○B.其它原因 ○C.视力及其它原因均有

*填写完该题，请跳至第 **22** 题。

15C、如果您最近有骑(驾)车，白天在熟悉的地方您在骑(驾)车时有多大困难? ( ) [单选题] *

○A. 没有困难 ○B.有一点困难 ○C.有困难(中等程度) ○D.的确很困难

16、在夜间骑(驾)车，您有多大困难? ( ) [单选题] *

○A.没有困难 ○B.有一点困难 ○C.有困难(中等程度)

○D.的确很困难 ○E.由于视力原因已不做此事 ○F.由于其他原因或没有兴趣而不做此事

16A、在困难条件下骑(驾)车，例例如在差的天气情况、交通高峰时段、高速公路路上或交通拥挤时，您有多大困难? ( ) [单选题] *

○A. 没有困难 ○B.有一点困难 ○C.有困难(中等程度)

○D.的确很困难 ○E.由于视力原因已不做此事 ○F.由于其他原因或没有兴趣而不做此事

17、由于视力原因，您是否经常不能完成预期的工作目标? ( ) [单选题] *

○A、一直是这样 ○B、大多数情况是这样 ○C、有时候是这样 ○D、偶尔是这样 ○E、没有

18、由于视力原因，您工作或做其它事情的时间是否受限( ) [单选题] *

○A、一直是这样 ○B、大多数情况是这样 ○C、有时候是这样 ○D、偶尔是这样 ○E、没有

19、是否经常出现由于眼睛及眼睛周围的疼痛、不适感而影响或妨碍到您想要做的事情？( ) [单选题] *

○A、一直是这样　○B、大多数情况是这样　○C、有时候是这样　○D、偶尔是这样　○E、没有

20、由于视力问题，我大多数时间等候待在家中( ) [单选题] *

○A、的确是这样　○B、大概是这样　○C、不确定　○D、大概不是这样　○E、确实不是这样

21、由于视力问题，我很多时间感到灰心丧气( ) [单选题] *

○A、的确是这样　○B、大概是这样　○C、不确定　○D、大概不是这样　○E、确实不是这样

22、由于视力问题，我很难对我所做的事情进行控制( ) [单选题] *

○A、的确是这样　○B、大概是这样　○C、不确定　○D、大概不是这样　○E、确实不是这样

23、由于视力问题，我不得不更更多地依赖他人告诉我很多事情( ) [单选题] *

○A、的确是这样　○B、大概是这样　○C、不确定　○D、大概不是这样　○E、确实不是这样

24、由于视力问题，我需要从别人那里得到更更多帮助( ) [单选题] *

○A、的确是这样　○B、大概是这样　○C、不确定　○D、大概不是这样　○E、确实不是这样

25、由于视力问题，我担心做出一些令自己或他人尴尬的事情( ) [单选题] *

○A、的确是这样　○B、大概是这样　○C、不确定　○D、大概不是这样　○E、确实不是这样

您的中文姓名 [填空题] *

____

您的年龄（请填写整数） [填空题] *

____

您的性别 [单选题] *

○男

○女

您是否有影响阅读的眼部疾病（如青光眼、白内障、800 度以上的高度近视） [单选题] *

○有（请具体填写）_________________ *

○无

您的左眼近视度数是多少度？ [填空题] *

____

您的右眼近视度数是多少度？ [填空题] *

____

您最近一月以来，平均每天的屏幕使用时长是多长时间？ [单选题] *

○A. <2h（电子屏使用很少，只在固定的时刻看手机电脑）

○B. 2-4h（很少使用电脑的轻度手机使用用户）

○C. 4-6h（使用电脑的轻松工作或者正常手机使用用户）

○D. 6-8h（正常工作或者中度手机使用用户，例如上网课）

○E. 8-10h（辛苦工作或者重度手机使用用户，例如上网课还要玩会儿手机）

○F. 10-12h（非常辛苦的工作，例如上一整天网课还要玩会儿手机）

○G. 12-14h（非常辛苦的工作，例如上一整天网课还要玩很久手机）

○H. 14-16h（除了睡觉的时间都在看电子屏幕）

○I. ＞16h（觉都不睡了）

您近一月来平均每天的手机使用时长是 [单选题] *

○A. 0(未使用手机)

○B. <2h(只在固定的时刻看手机，使用很少)

○C. 2-4h(轻度手机使用用户)

○D. 4-6h(中度手机使用用户)

○E. 6-8h(重度手机使用用户)

○F. 8-10h(需要使用手机工作或者非常重度的手机使用用户)

○G. 10-12h(需要使用手机进行非常辛苦的工作，例如上一整天网课还要玩会儿手机)

○H. ＞12h(几乎除了睡觉的时间都在使用手机)

您近一月来平均每天的电脑使用时长是 [单选题] *

○A. 0(未使用电脑)

○B. 0-2h(很少使用电脑)

○C. 2-4h(轻度电脑使用用户，例如需要使用电脑完成单项任务)

○D. 4-6h(正常电脑使用用户，例如需要使用电脑工作)

○E. 6-8h(中度电脑使用用户，例如白天需要使用电脑工作)

○F. 8-10h(重度电脑使用用户，白天和夜间都需要使用电脑工作)

○G. 10-12h(需要使用电脑进行非常辛苦的工作，例如上一整天网课还要玩会儿游戏)

○H. ＞12h(几乎除了睡觉的时间都在使用电脑)

您近一月来平均每天的平板电脑或 pad 使用时长是 [单选题] *

○A. 0(未使用平板或 pad)

○B. 0-2h(轻度使用平板或 pad)

○C. 2-4h(中度平板或 pad 使用用户，例如需要使用平板或 pad 完成单项任务)

○D. 4-6h(中度平板或 pad 使用用户，例如需要使用平板或 pad 工作)

○E. 6-8h(重度平板或 pad 使用用户，例如使用平板或 pad 追剧)

○F. 8-10h(重度平板或 pad 使用用户，白天和夜间都需要使用平板或 pad)

○G. 10-12h(整日使用平板或 pad，例如上一整天网课还要玩会儿游戏)

○H. ＞12h(几乎除了睡觉的时间都在使用平板或 pad)

您的学历是 [单选题] *

○高中及以下（含在读）

○本科（含在读）

○硕士研究生（含在读）

○博士研究生（含在读）

○博士后（含在读）
