# Supplementary material for: Using the C-Read as a Portable Device to Evaluate Reading Ability in Young Chinese Adults: An Observational Study
Source: J Pers Med. 2023 Mar 1;13(3):463. doi: 10.3390/jpm13030463 (PMC10056310; doi:10.3390/jpm13030463)
Supplement: Supplementary file 1 [file jpm-13-00463-s001.zip › supplementary file.pdf]

Table S1. Parameter Estimates for MLR Model for RA, MRS and CPS in right, left and both eyes.

Measure			Multivariate Linear Regression		Stepwise Regression (p value = 0.05)	
			β (95% CI)	p value	β (95% CI)	p value
RA	right eye	Intercept	0.23(0.10 to 0.36)	<0.001	0.21(0.17 to 0.25)	<0.001
		Age,y	-0.00065(-0.0053 to 0.0040)	0.781		
		Screentime, h	-0.00076(-0.014 to 0.012)	0.908		
		Myopia,D	-0.012(-0.021 to -0.002)	0.015	-0.12(-0.021 to -0.003)	0.013
	left eye	Intercept	0.19(0.08 to 0.31)	0.001	0.22(0.18 to 0.26)	<0.001
		Age,y	-0.00084(-0.0051 to 0.0035)	0.700		
		Screentime, h	0.0095(-0.0028 to 0.022)	0.128		
		Myopia,D	-0.012(-0.205 to -0.004)	0.006	-0.013(-0.021 to -0.005)	0.003
	both eyes	Intercept	0.213(0.128 to 0.297)	<0.001	0.22(0.19 to 0.24)	<0.001
		Age,y	-0.00074(-0.00387 to 0.00239)	0.642		
		Screentime, h	0.0044(-0.0045 to 0.0133)	0.331		
		Myopia,D	-0.012(-0.018 to -0.006)	<0.001	-0.012(-0.018 to -0.006)	<0.001
MRS	right eye	Intercept	158.92(117.71 to 200.12)	<0.001	183.82(177.53 to 190.10)	<0.001
		Age,y	0.40(-1.10 to 1.89)	0.602		
		Screentime, h	2.12(-2.10 to 6.34)	0.323		
		Myopia,D	-1.33(-4.32 to 1.67)	0.383		
	left eye	Intercept	196.09(159.69 to 232.50)	<0.001	189.91(153.78 to 225.06)	<0.001
		Age,y	-1.38(-2.74 to -0.01)	0.049	-1.39(-2.78 to -0.01)	0.048
		Screentime, h	4.99(1.08 to 8.91)	0.013	4.47(0.56 to 8.38)	0.026
		Myopia,D	2.57(-0.13 to 5.27)	0.062		
	both eyes	Intercept	179.77(152.39 to 207.15)	<0.001	166.0(150.9 to 181.1)	<0.001
		Age,y	-0.55(-1.56 to 0.47)	0.289		
		Screentime, h	3.45(0.57 to 6.33)	0.019	3.19(0.34 to 6.04)	0.028
		Myopia,D	0.68(-1.33 to 2.69)	0.507		
CPS	right eye	Intercept	0.295(0.147 to 0.443)	0.048	0.35(0.33 to 0.38)	<0.001
		Age,y	-0.004(-0.010 to 0.002)	0.47		
		Screentime, h	0.012(-0.004 to 0.027)	0.010		
		Myopia,D	-.00087(-0.020 to 0.002)	0.118		
	left eye	Intercept	0.26(0.13 to 0.40)	<0.001	0.32(0.28 to 0.36)	<0.001
		Age,y	-0.003(-0.008 to 0.002)	0.516		
		Screentime, h	0.014(0.001 to 0.028)	0.300		
		Myopia,D	-0.013(-0.023 to -0.003)	0.006	-0.014(-0.024 to -0.004)	0.004
	both eyes	Intercept	0.19(0.09 to 0.30)	<0.001	0.23(0.16 to 0.29)	<0.001
		Age,y	-0.0014(-0.024 to 0.005)	0.467		
		Screentime, h	0.015(0.004 to 0.026)	0.010	0.015(0.004 to 0.026)	0.007
		Myopia,D	-0.013(-0.021 to -0.006)	<0.001	-0.013(-0.021 to -0.006)	<0.001

Abbreviations: D, diopters; RA, reading acuity; MRS, maximum reading speed; CPS, critical print size

Table S2. VFQ-25 Sub-scales Scores of Participants in This Study

Characteristic	General Vision	Ocular Pain	Near Activities	Distance Activities	Social Function	Mental Health	Role Difficulties	Dependency	Driving	Color Vision	Peripheral Vision
VFQ-25	67.43 (13.64)	85.61 (14.40)	93.27 (12.67)	88.04 (13.64)	95.53 (11.70)	83.77 (15.67)	90.37 (15.26)	93.27 (15.26)	92.79 (10.69)	96.33 (13.92)	91.40 (16.38)

Abbreviations: VFQ, visual functioning questionnaire

Table S3. VFQ-25 scores in different gender, education, and myopia

	**Gender**		**Education**			**Myopia**			
Characteristic	male (n = 116)	female (n = 91)	Undergraduate (n = 133)	Postgraduate (n = 26)	Doctor & Postdoc (n = 30)	no (n = 40)	low (n = 144)	moderate (n = 187)	high (n = 43)
VFQ-25	86.95 (9.24)	85.37 (8.93)	86.48 (8.82)	87.72 (7.61)	83.95 (11.37)	88.17 (6.54)	86.86 (9.49)	85.83 (9.59)	84.34 (7.21)
P value	0.22		0.24			0.19			

Abbreviations: VFQ, visual functioning questionnaire

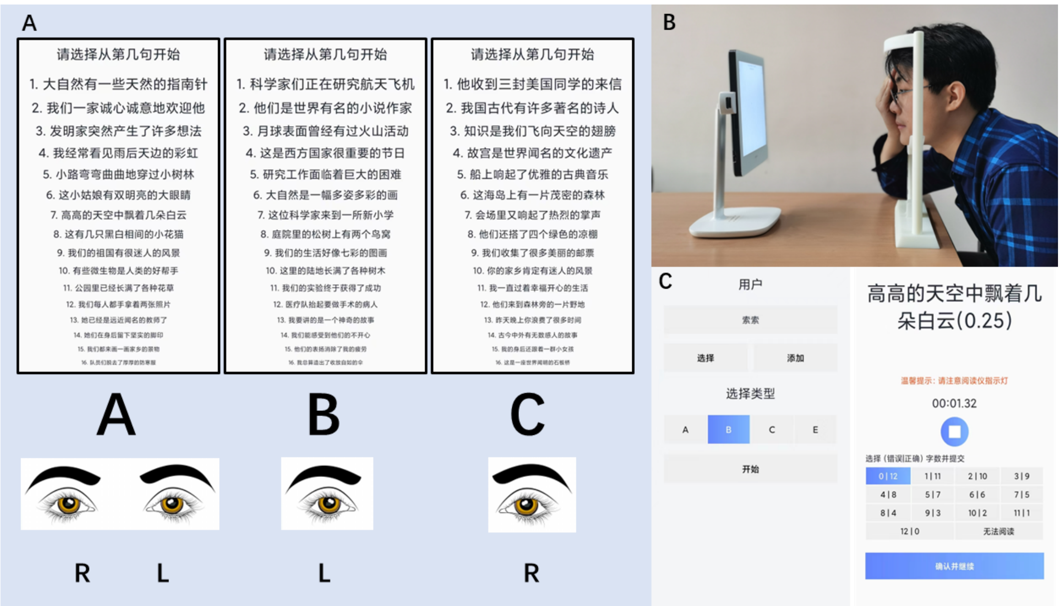

Figure S1. A brief introduction to the C-Read system. **a**, There are three different scales, A, B, C, in C-Read system to assess participants' binocular, right-eye, and left-eye reading ability, respectively. Each scale consists of 16 Chinese sentences and each sentence consisting of 12 simplified Chinese characters. The length and content of the sentences are carefully selected according to the characteristics of the Chinese language (e.g. sentences with subordinate clauses are not selected, and each sentence contains the same number of simple or complex characters), ensuring that the test tool is adapted to the specific needs of Chinese reading tests. **b,** An actual C-Read test: a participant is undergoing a C-Read examination under the supervision of an ophthalmologist. **c**, The operating end of C-Read system. The examiner can switch to a different scale for the next test and record the participant's reading time using the C-Read application on the smartphone.

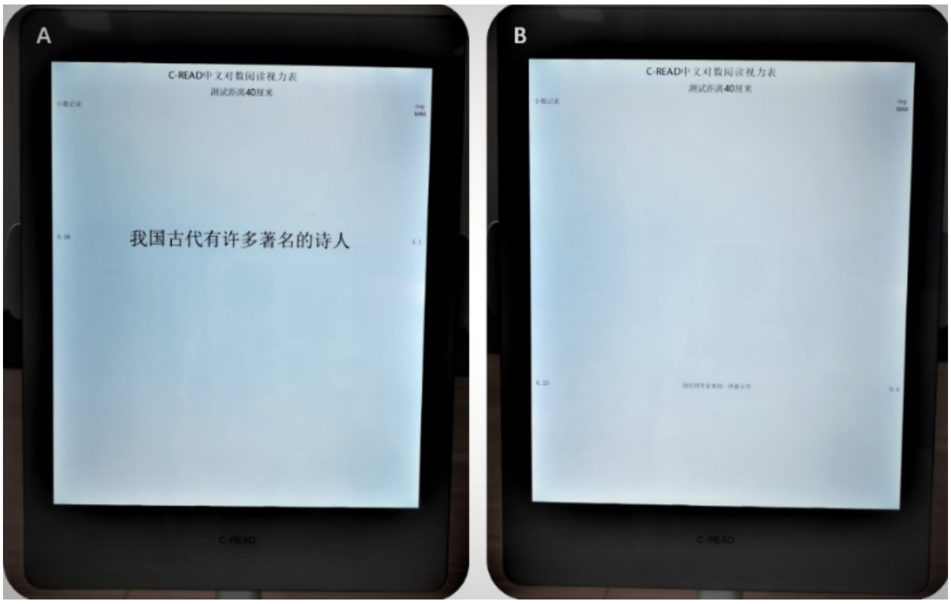

Figure S2. The screen of C-Read device. **a,** The C-Read system includes a high-definition screen with intelligent display and the ability to synchronize with smartphone application via Bluetooth. The device has a voice recording function and is able to store test data and record patient details. **b,** One sentence from another C-Read scale. **S**pecific Chinese sentences are displayed on the screen during the test and the participant is guided to read them aloud. The font size of the sentences decreases from the top to the bottom. Each time the screen will only display one sentence.

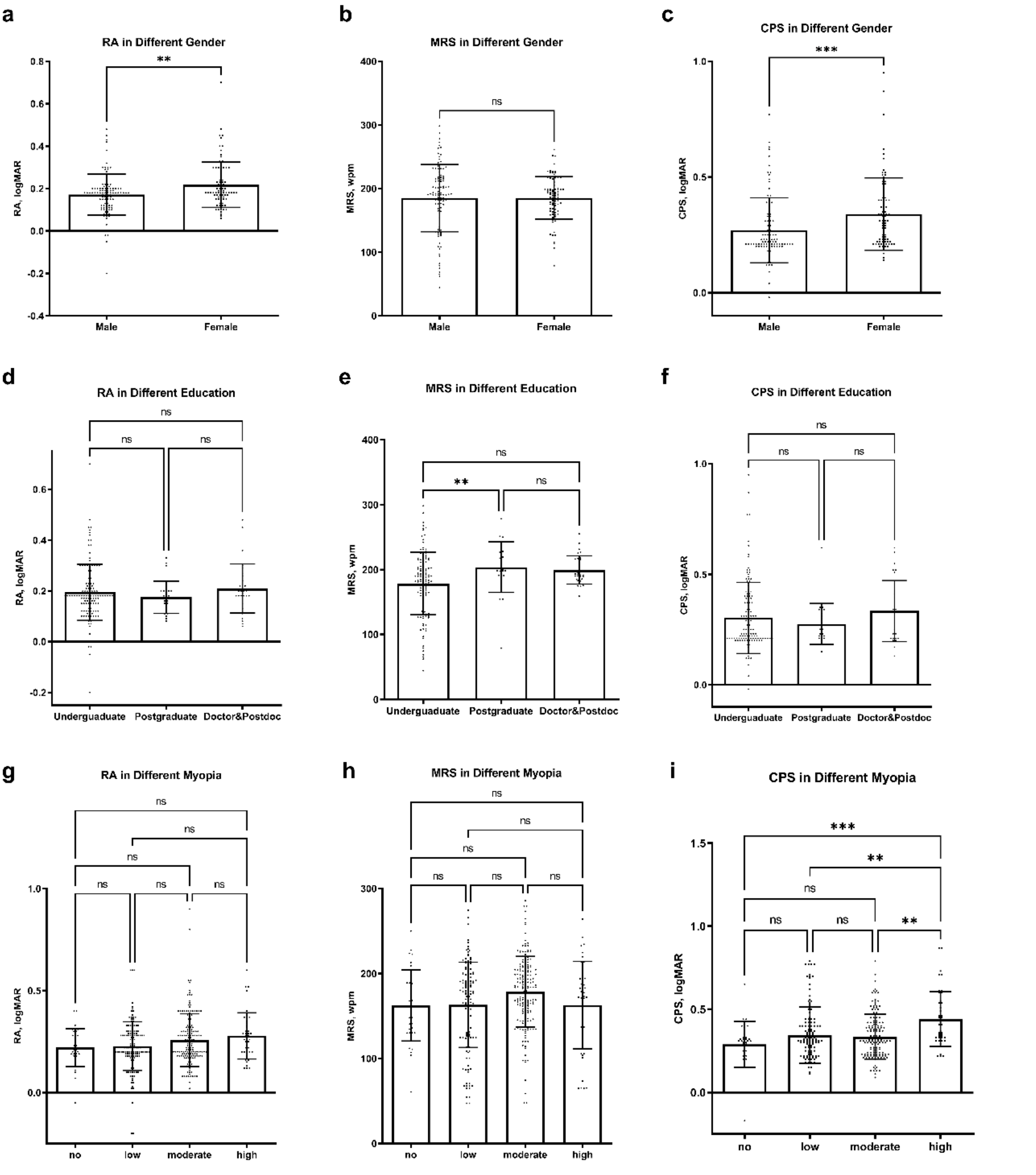

Figure S3. Reading characteristics for left & right eyes in the different sexes, education levels, and myopia levels. a, The RA in different sexes was statistically significant (n = 191, p = 0.009), as determined by the two-tailed Mann Whitney test. b, The MRS in different sexes was not statistically different (n = 191, p = 0.490), as determined by the two-tailed Mann Whitney test. c, The CPS in the different sexes was statistically significant (n = 191, p < 0.001), as determined by the two-tailed Mann Whitney test. d, The RA of the three education levels was not significantly different (n = 189, p = 0.252), as determined by Kruskal-Wallis test. e, The MRS in the three education levels was statistically significant (n = 189, p = 0.003), as determined by

Kruskal-Wallis test. f, The CPS in the different education levels was not statistically different (n = 189, p = 0.394), as determined by Kruskal-Wallis test. g, The RA in the different myopia levels was not statistically significant (n = 382 eyes, p = 0.157), as determined by Kruskal-Wallis test. h, The MRS in different myopia levels was statistically significant (n = 382 eyes, p = 0.039), as determined by Kruskal-Wallis test. i, The CPS in different myopia levels was statistically significant (n = 382 eyes, p < 0.001), as determined by Kruskal-Wallis test. Abbreviations: RA (reading acuity), MRS (maximum reading speed), CPS (critical print size). Significance: ns, $p > 0.05$; *$p < 0.05$; **$p < 0.01$; ***$p < 0.001$.

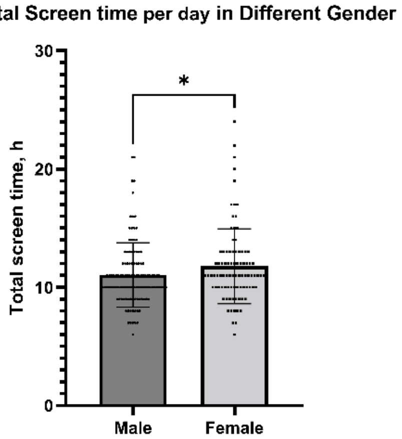

Figure S4. Total screen time per day in different gender. The total screen time in different sexes was statistically different (n = 207, $p = 0.04$), as determined by Mann Whitney test. Significance: *$p < 0.05$

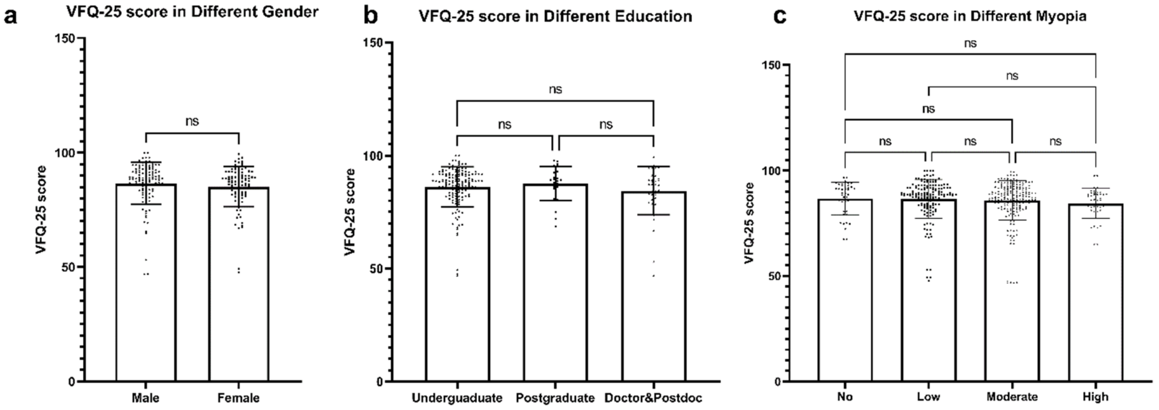

Figure S5. VFQ-25 scores in different gender, education, and myopia. **a**, The VFQ-25 scores in different sexes was not statistically different (n = 207, $p = 0.07$), as determined by Mann Whitney test. **b**, The VFQ-25 scores in different education was not statistically different (n = 189, $p = 0.40$), as determined by Kruskal-Wallis test. **c**, The VFQ-25 scores in different myopia was not statistically different (n = 414 eyes, $p = 0.06$), as determined by Kruskal-Wallis test. Abbreviations: VFQ (visual functioning questionnaire). Significance: ns, $p > 0.05$.
